# IL-4 Is a Key Requirement for IL-4- and IL-4/IL-13-Expressing CD4 Th2 Subsets in Lung and Skin

**DOI:** 10.3389/fimmu.2018.01211

**Published:** 2018-06-01

**Authors:** Melanie Sarah Prout, Ryan L. Kyle, Franca Ronchese, Graham Le Gros

**Affiliations:** Malaghan Institute of Medical Research, Wellington, New Zealand

**Keywords:** Th2, IL-4, IL-13, skin, lymph node, lung, non-lymphoid tissues

## Abstract

Although IL-4 is long associated with CD4 Th2 immune responses, its role in Th2 subset development in non-lymphoid tissues is less clear. We sought to better define IL-4’s role in CD4 Th2 responses by using transgenic mice that express a dual IL-4 AmCyan/IL-13 DsRed (IL-4AC/IL-13DR) fluorescent reporter on an IL-4-sufficient or IL-4-deficient background. Using primary Th2 immune response models against house dust mite or *Nippostrongylus brasiliensis* (*Nb*) allergens, we examined the requirement for IL-4 by each of the defined Th2 subsets in the antigen draining lymph node, skin, and lung tissues. In the lymph node, a CXCR5^+^PD-1^+^ T follicular helper (Tfh) and a CXCR5^lo^PD-1^lo^ Th2 subset could be detected that expressed only IL-4AC but no IL-13DR. The number of IL-4AC^+^ Tfh cells was not affected by IL-4 deficiency whereas the number of IL-4AC^+^ Th2 cells was significantly reduced. In the non-lymphoid dermal or lung tissues of allergen primed or *Nb*-infected mice, three strikingly distinct T cell subsets could be detected that were IL-4AC, or IL-4AC/IL-13DR, or IL-13DR CD4. The IL-4- and IL-4/IL-13-expressing subsets were significantly reduced in IL-4-deficient mice, while the numbers of IL-13-expressing CD4 T cells were not affected by IL-4 deficiency indicating that other factors can play a role in directing the development of this Th2 subtype. Taken together, these data indicate that the appearance of IL-4-expressing Tfh cells in the lymph node is not dependent on IL-4 while the appearance of IL-4-expressing Th2 subsets in the lymph node and IL-4, IL-4/IL-13-expressing Th2 subsets in skin and lung tissues of antigen primed mice is significantly IL-4 dependent.

## Introduction

The cytokine IL-4 is understood to be key to the development of type 2 immune responses that underlie allergic disease pathologies and immunity to parasites; however, the specific role IL-4 plays in CD4 T cell differentiation is less clear. Early investigations using *in vitro* culture systems revealed the dominant role that IL-4 plays in driving and shaping CD4 Th2 subset differentiation toward expression of the canonical type 2 cytokines IL-4, IL-5, and IL-13 (1, 2). Although *in vivo* studies have indicated a role for IL-4 directing Th2 development in the lymph node (3, 4), further studies revealed a more subtle role for IL-4 with IL-4-expressing Th2 cells appearing in the draining lymph nodes of immunized mice seemingly independent of IL-4 or STAT6 signaling (5, 6). Other studies have shown that the level of TCR activation can play a role in Th2 differentiation (7), while recent studies even support the view that Th2 development occurs in the tissues and is fully regulated by tissue-specific checkpoints (8). In addition, TSLP elicited basophils have been shown to promote epicutaneous sensitization to food antigens and subsequent IgE mediated food allergy through IL-4 (9).

We took the opportunity to clarify the role of IL-4 in *in vivo* CD4 Th2 subset differentiation by asking whether certain specific Th2 subsets are more sensitive to the influence of IL-4 and whether IL-4-is required for Th2 subset development at non-lymphoid tissue sites such as the skin and lung. For the *in vivo* immune response studies, we used our recently developed antigen priming ear model (10) to quantitatively analyze the character, kinetics and magnitude of the IL-4- and IL-13-producing Th2 subsets that appear in the ear and ear draining lymph node following allergen priming (11, 12). To examine the appearance Th2 subsets in the lung, we used *Nippostrongylus brasiliensis* infection model which involves a lung migration stage in its infection cycle (13). In following the appearance of IL-4- and IL-13-expressing Th2 subsets, we were concerned to reduce artifact and bias inherent in restimulation and intracellular cytokine staining techniques and previously reported reporter IL-4 knockout mice (14–16). Therefore, we used the validated sensitivity of the recently developed *Il4* and *Il13* transcriptional reporter 4C13R mice (17) to investigate the appearance of IL-4-AmCyan (IL-4AC)- and IL-13-DsRed (IL-13DR)-expressing CD4^+^ T cells arising in both the lymphoid and non-lymphoid tissues responding to house dust mite (HDM) or *Nippostrongylus brasiliensis* (*Nb*) allergens in either an IL-4-sufficient or -deficient environment. The *Il4*AC/*Il13*DR reporter BAC transgene construct is independent of the endogenous *Il4/Il13* locus in the mouse and appears able to faithfully report the commitment of CD4 T cells to the expression of the canonical type 2 cytokine gene expression pattern (17–19) under the appropriate tissue culture and relevant *in vivo* immunization protocols without affecting normal type 2 immune effector functions (20). We were able to detect both IL-4-expressing Tfh and Th2 cells in the draining lymph nodes of HDM challenged mice and while the small number of IL-4AC Th2 cells was significantly reduced by removal of IL-4, the Tfh cells were independent of the need for IL-4. Strikingly, analysis of the CD4 T cells that migrated to the skin 7 days after the allergen challenge revealed three functionally distinct Th2 subsets that could be defined by their cytokine expression patterns, IL-4AC, IL-4AC/IL-13DR, and IL-13DR only. The appearance of the IL-4AC- and IL-4AC/IL-13DR-expressing Th2 cell subsets was highly dependent on IL-4 while the IL-13DR Th2 subset was not affected by the IL-4-deficient background. Taken together, our findings reveal the fundamental role that IL-4 plays in the development of functionally diverse effector Th2 subsets in tissues and lymph node. We also identify a novel IL-13-producing CD4^+^ Th2 subset that appears in the skin following allergen challenge and does not require IL-4 for expression of IL-13.

## Materials and Methods

### Mice

4C13R (17) reporter mice were bred and maintained on a C57BL/6 background in the Malaghan Institute of Medical Research Biomedical Research Unit. The *Il4*AmCyan/*Il13*DsRed construct does not interfere with normal immune function with similar levels of T cells, B cell, immunoglobulin, and inflammatory cells being induced by immunization compared with wild-type C57BL/6 mice. To generate IL-4-deficient reporter strains, 4C13R mice were crossed with IL-4^G4/G4^ mice (6) to generate 4C13R × IL-4^G4/+^ (IL-4^+/−^) mice and 4C13R × IL-4^G4/+^ mice were crossed with IL-4^G4/G4^ mice to generate 4C13R × IL-4^G4/G4^ (IL-4^−/−^) mice. All animal procedures were approved by the Victoria University of Wellington Animal Ethics committee and performed in accordance with institutional guidelines.

### Ear Immunizations

Mice were anesthetized using xylazine and ketamine (Phoenix, New Zealand). 30 µl of a solution containing 200 µg HDM (Greer Laboratories, Lenoir, NC, USA) or 600 dead L3 *Nb* was injected into the ear pinnae as described (10).

### *Nb* Infection

Mice were inoculated with 550 L3 *Nb* larvae by s.c. injection.

### Cell Isolation

All tissues were isolated 7 days posttreatment. Auricular draining lymph nodes were pressed through 70-μm cell strainers into complete media [IMDM (GIBCO) supplemented with 5% FBS (Sigma), 100 U/ml penicillin/100 μg/ml streptomycin (Invitrogen), and 55 µM β-mercaptoethanol (Invitrogen)] to make a single-cell suspension. Dorsal and ventral ear sheets were separated and minced into very fine pieces using scissors. Each ear was digested by incubating for 30 min at 37°C with shaking, in ear digestion mix [IMDM (GIBCO) containing 1.2 mg/ml Collagenase IV (Sigma) and 120 μg/ml DNAse I (Roche)]. Solution was pipetted up and down and passed through 70-μm cell strainers into Ear wash buffer [PBS containing 1% BSA (Sigma), 5 mM EDTA (Invitrogen), and 120 μg/ml DNAse I (Roche)]. Cells were washed once more in Ear wash buffer before resuspension in cIMDM. Bronchoalveolar lavage (BAL) was performed by cannulation of mice and washing airways three times with PBS. Lungs were finely minced and digested in lung digestion mix [IMDM (GIBCO) containing 2.4 mg/ml Collagenase I (GIBCO) and 120 μg/ml DNAse I (Roche)] for 1 h at 37°C. Live cell counts performed using a hemocytometer and trypan blue (Invitrogen) exclusion.

### *In Vitro* Restimulation

Day 7 ear lymph node cells (1 × 10^6^/well) were cultured for 19 h on plates coated with 1 μg/ml anti-CD3 (145-2C11) with cIMDM, 100 U/ml rIL-2, and 1/50 dilution of anti-CD28 (37.51) supernatant corresponding to 5 μg/ml.

### FACS Analysis

Cells were resuspended in a buffer containing 0.01% NaN_3_ (Sigma), 2% FBS, and 2 mM EDTA (Life Technologies) in PBS then incubated with anti-CD16/32 antibody (clone 2.4G2) before staining with fluorophore-conjugated antibodies. Cells were stained with antibodies against the following molecules (clone, conjugate; source): CD45 (30-F11, APC-Cy7, BD), CD3 (145-2C11, BV786; BD), CD3 (145-2C11, BUV395; BD), CD4 (RM4-5, BV605; BD), CD4 (GK1.5, APC-Cy7; BD), CD8 (53-6.7, AF700; BioLegend), TCRγδ (GL3, PE-Cy7; BioLegend), CD44 (IM7, APC; BD), CXCR5 (2G8, Biotin; BD), and PD-1 (RMP1-30, PerCP-ef710; eBioscience). Streptavidin-BV605 (BD) was also used. Stained cells were resuspended in viability dye DAPI to exclude dead cells. IL-4 and IL-13 were detected by the IL-4AC and IL-13DR reporters in 4C13R mice. Data were acquired on a BD LSRFortessa SORP flow cytometer (Becton Dickinson, San Jose, CA, USA). Flow data were analyzed using FlowJo software (Treestar).

### Statistical Analysis and Graphics

Statistical analyses were performed using GraphPad Prism software (GraphPad Software Inc., San Diego, CA, USA). Statistical comparisons used are specified in figure legends.

## Results

### Priming With HDM Allergen Induces the Development of Th2 Subsets Producing Only IL-4AC in the Lymph Node and Distinct IL-4AC^+^, IL-4AC^+^IL-13DR^+^, or IL-13DR^+^ Expression Profiles in the Ear Tissue

**Figure 1:**
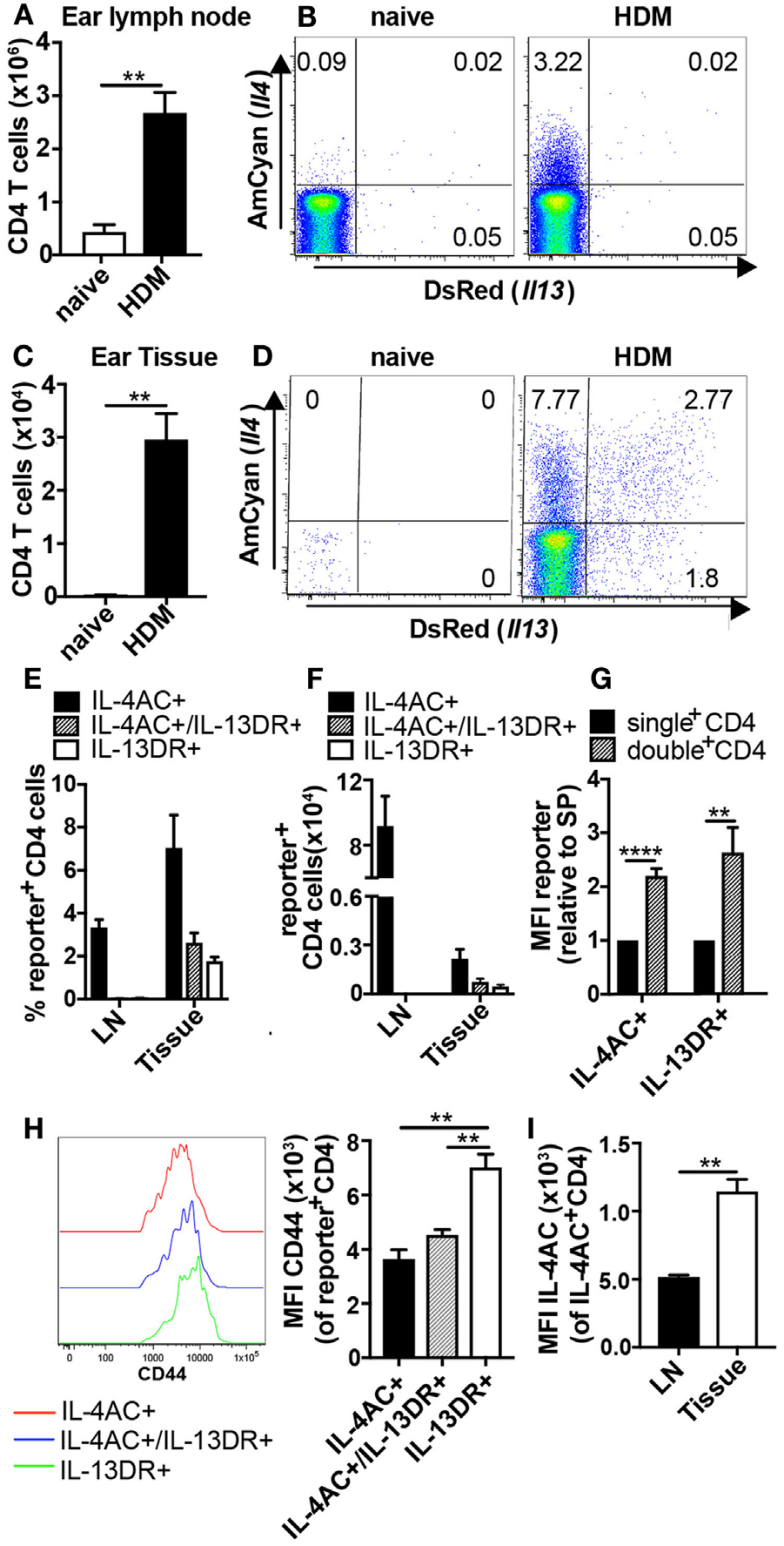
Priming with house dust mite (HDM) allergen induces the development of Th2 subsets producing only IL-4AC in the lymph node and distinct IL-4AC, IL-4AC/IL-13DR, or IL-13DR expression profiles in the ear tissue. 4C13R transgenic mice were challenged with 200 μg HDM i.d. in the ear. Ear draining lymph nodes and ear tissue were harvested 7 days later, and the presence of IL-4AC- and IL-13DR-expressing CD4 Th2 cells examined by flow cytometry. **(A,C)** Number of CD4 T cells in ear lymph node and ear tissue. **(B,D)** Concatenated FACS plots of CD4 T cells from naïve and HDM primed 4C13R transgenic mice showing IL-4AC^+^ and IL-13DR^+^ cells. **(E)** Proportions and **(F)** numbers of IL-4AC^+^, IL-4AC^+^/IL-13DR^+^, and IL-13DR^+^ subsets in the ear lymph node and ear tissue. **(G)** Median fluorescent intensity (MFI) of IL-4AC and IL-13DR reporters expressed in single reporter^+^ vs double reporter^+^ CD4 Th2 cells in the ear tissue (relative to MFI of single-positive cells). **(H)** MFI of CD44 expression on IL-4AC^+^, IL-4AC^+^/IL-13DR^+^, and IL-13DR^+^CD4^+^ Th2 cells in the ear tissue. 96–98% of the reporter positive Th2 cells were CD44^+^ **(I)** MFI of IL-4AC expression in IL-4AC^+^ CD4 cells from ear lymph node and ear tissue. **(A–G,I)** Data from an experiment (*n* = 3–5) representative of nine lymph node experiments and seven ear tissue experiments. **(H)** Results from a single experiment (*n* = 3), representative of three experiments. Data show mean + SEM (**p ≤ 0.01 and ****p ≤ 0.0001 two-tailed *t*-test).

To follow the activation, differentiation, and migration patterns of allergen-induced Th2 subsets, 4C13R mice were primed intradermally (i.d.) in the ear pinnae with HDM. The kinetic studies showed IL-4AC^+^ CD4 T cells to be maximally elevated in the ear draining lymph node on days 5–9 after priming (Figures S1A,B in Supplementary Material), therefore ear lymph nodes and ear tissue were examined on day 7 for the appearance of IL-4AC- and IL-13DR-expressing CD4 T cells. CD4 T cells were defined as CD45^+^CD3^+^CD4^+^CD8^−^TCRγδ^−^ cells (Figures S2A,B in Supplementary Material), thus excluding any TCRγδ^+^ intraepithelial lymphocytes found in the ear tissue. HDM priming resulted in significant infiltration of CD4 T cells into these sites over the 7-day period, with a sixfold increase in the lymph node to 2.6 × 10^6^ CD4 cells, while immunized ears had 2.9 × 10^4^ CD4 cells compared with very few in naive mice (Figures 1A,C). The lymph node and ear tissue CD4^+^ T cells displayed distinctly different Th2 cytokine profiles, which we could observe using the *Il4* and *Il13* gene locus directed expression of the AmCyan and DsRed fluorescent reporters, respectively (Figures 1B,D–F). In the ear draining lymph node, only IL-4AC-expressing CD4 Th2 cells could be detected, whereas in the ear tissue three distinct CD4 T cell subpopulations were found; IL-4AC single positives, IL-4AC/IL-13DR double positives and IL-13DR single positives. Comparison of the reporter median fluorescent intensities (MFIs) for each of these ear tissue-derived CD4 T cell subsets reveals that the double-positive CD4 Th2 cells have a higher MFI for both IL-4AC and IL-13DR reporters than do the single positives (Figure 1G), reflecting a higher degree of activation and the potential for producing a greater amount of cytokine on a per cell basis. As reported previously (6) *in vivo* generated IL-4 reporter positive cells expressed high levels of CD44, interestingly the IL-13DR single-positive CD4 T cells demonstrated the highest MFI level for CD44 expression (Figure 1H), indicating they had a greater degree of activation than the IL-4AC- or IL-4AC/IL-13DR-expressing cells. Interestingly the tissue sourced IL-4AC^+^ CD4 cells have a greater MFI of IL-4AC potentially reflecting their potential for higher levels of gene expression from the *Il4* locus than those CD4 T cells sourced from the lymph node (Figure 1I). Priming of mice with dead L3 *Nb* larvae in the ear stimulated a similar pattern of IL-4AC and IL-13DR expression by CD4 T cells in lymph node and tissues to that seen in HDM primed mice (Figures S3A–G in Supplementary Material).

To determine if the IL-4AC-expressing Th2 cells in the ear lymph node have the potential to produce IL-13DR upon *in vitro* restimulation, ear lymph node cells were harvested 7 days post HDM priming, and the whole lymph node cell population was cultured on anti-CD3 for 19 h. Stimulated CD4 cells expressed a similar proportion of IL-4AC as they did *ex vivo* (2.7%), while the percentage of CD4 T cells expressing IL-4AC cultured without anti-CD3 reduced over the time of culture (1.5%). Restimulation of the CD4 T cell subsets did not induce any further IL-13DR expression above background levels, indicating that the failure to detect IL-13DR expression in the lymph node was not due to lack of activation but rather the level of differentiation of the IL-4AC^+^ CD4 T cells (Figures S4A–C in Supplementary Material).

These data demonstrate that following allergen priming in the ear tissue, distinct Th2 subsets develop in the draining lymph node and tissues. Specifically, CD4 T cells become committed to IL-4AC expression in both lymph node and tissues while the commitment of CD4 T cells to IL-13DR expression only occurs in the tissue.

### Both Tfh Cells and Th2 Cells Contribute to IL-4 Expression in the Lymph Node After HDM Challenge

**Figure 2:**
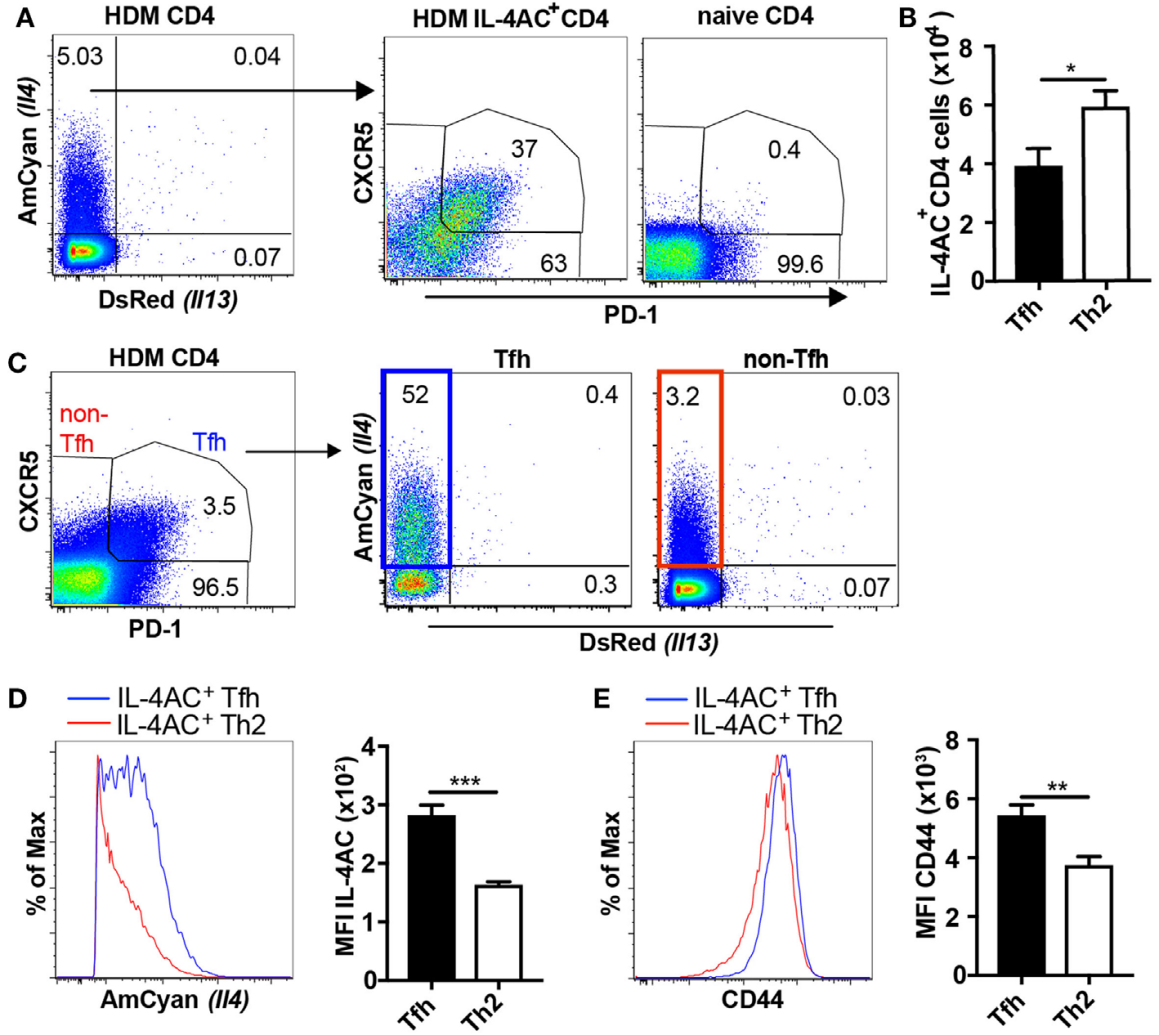
Both T follicular helper (Tfh) cells and Th2 cells contribute to IL-4AC expression in the lymph node after house dust mite (HDM) challenge. 4C13R transgenic mice were treated with 200 μg HDM i.d. in the ear. The ear draining lymph nodes were harvested 7 days posttreatment and analyzed by flow cytometry. **(A)** FACS plots showing the proportion of IL-4AC^+^ CD4 T cells and then the proportion of CXCR5^+^PD-1^+^ Tfh and CXCR5^lo^PD-1^lo^, CXCR5^+^PD-1^lo^, and CXCR5^lo^PD-1^+^ Th2 cells within this population. **(B)** Numbers of IL-4AC^+^ Tfh and IL-4AC^+^ Th2 CD4 T cells. **(C)** FACS plots showing the proportion of CXCR5^+^PD-1^+^ Tfh and CXCR5^lo^PD-1^lo^, CXCR5^+^PD-1^lo^, and CXCR5^lo^PD-1^+^ non-Tfh CD4 T cells and then the proportion of IL-4AC^+^ CD4 T cells within these populations. Median fluorescent intensity (MFI) of IL-4AC **(D)** and CD44 **(E)** expression on IL-4AC^+^ Tfh and IL-4AC^+^ Th2 CD4 T cells. **(A,C–E)** Data from a representative experiment (*n* = 6) of four experiments. **(B)** Data pooled from four experiments (*n* = 23). Data show mean + SEM (*p ≤ 0.05, **p ≤ 0.01, and ***p ≤ 0.001 two-tailed *t*-test).

As many studies have shown T follicular helper (Tfh) cells to be a dominant source of IL-4 in the lymph node (21–23), we sought to clarify the identity of the IL-4-expressing CD4 T cells observed in the ear lymph nodes of HDM primed mice. In analyzing the lymph node-derived IL-4AC^+^ CD4 T cells, we identified both CXCR5^+^PD-1^+^CD4^+^ (Tfh) and CXCR5^lo^PD-1^lo^, CXCR5^+^PD-1^lo^, and CXCR5^lo^PD-1^+^CD4^+^ (Th2) populations (Figure 2A). Approximately 40% of the IL-4AC^+^ cells were Tfh cells as defined by PD-1 and CXCR5 expression while the remaining were Th2 cells (Figure 2B). Gating on IL-4AC^+^ Tfh and IL-4AC^+^ Th2 cells as shown in Figure 2C allowed us to compare these two populations, showing the IL-4AC^+^ Tfh cells to have a higher level of IL-4AC expression compared with the IL-4AC^+^ Th2 cells (Figure 2D). In addition, the IL-4AC^+^ Tfh cells showed a higher level of CD44 expression than the IL-4AC^+^ Th2 cells (Figure 2E). The biological impact of this statistically significant difference is unclear.

Thus, it would appear from these data that following HDM introduction to the skin, CD4 T cells in the draining lymph node become committed to activated Tfh and Th2 phenotypes some of which express IL-4 and contribute to IL-4 production in the ear draining lymph node. Interestingly, Tfh cells also expressed higher levels of the IL-4AC reporter, perhaps suggesting a higher capacity to secrete IL-4.

### IL-4 Plays a Role in the Development of IL-4AC-Expressing Lymph Node Th2 Cells but Not IL-4AC-Expressing Tfh Cells

**Figure 3:**
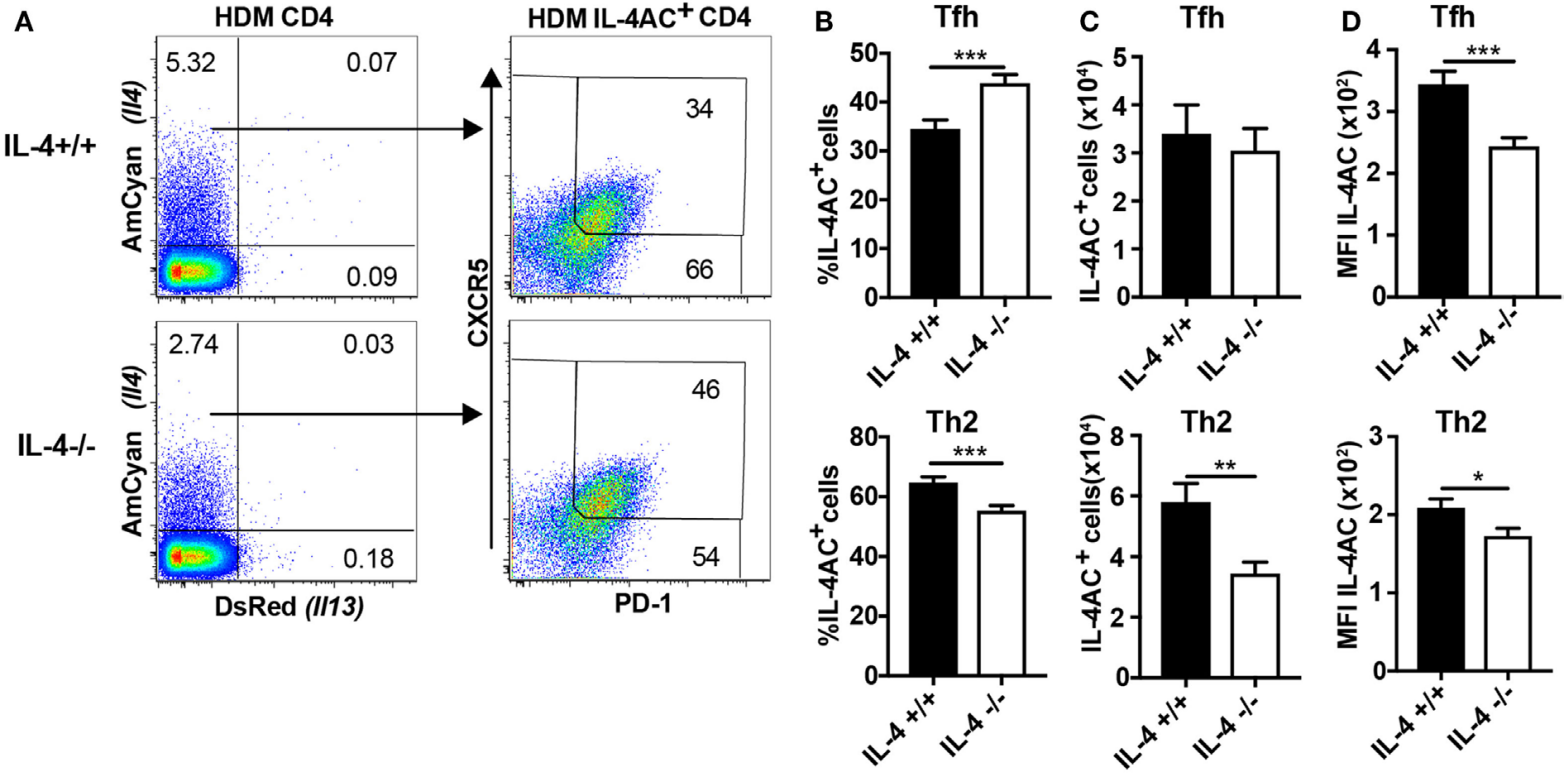
IL-4 plays a role in the development of IL-4AC-expressing lymph node Th2 cells but not IL-4AC-expressing T follicular helper (Tfh) cells. 4C13R-IL-4^+/+^ and 4C13R-IL-4^−/−^ mice were treated with 200 μg house dust mite (HDM) i.d. in the ear. The ear draining lymph nodes were harvested from mice 7 days posttreatment, and lymph nodes were analyzed by flow cytometry. **(A)** FACS plots showing the proportion of IL-4AC^+^ CD4 T cells and then the proportion of CXCR5^+^PD-1^+^ Tfh and CXCR5^lo^PD-1^lo^, CXCR5^+^PD-1^lo^, and CXCR5^lo^PD-1^+^ Th2 cells within this population. **(B)** Proportion of IL-4AC^+^CD4 T cells that are Tfh or Th2 cells. **(C)** Numbers of IL-4AC^+^ Tfh and IL-4AC^+^ Th2 CD4 T cells and **(D)** median fluorescent intensity (MFI) of IL-4AC of IL-4AC^+^ Tfh and IL-4AC^+^ Th2 CD4 T cells. **(A)** FACS plots from a representative experiment (*n* = 6). **(B,C)** Data pooled from three experiments (*n* = 18). **(D)** Data from a representative experiment (*n* = 6). Data show mean + SEM (*p ≤ 0.05, **p ≤ 0.01, and ***p ≤ 0.001 two-tailed *t*-test).

We next sought to investigate the role of IL-4 in the development of IL-4AC-expressing CD4 T cell populations in the draining lymph nodes of mice primed i.d. with HDM allergen. CD4 T cells from HDM primed IL-4^−/−^ mice had a reduced proportion of IL-4AC-expressing CD4 cells compared with IL-4^+/+^ mice (Figure 3A). Distinguishing Tfh and Th2 populations within the IL-4AC^+^ cell subset reveals a shift in the proportion of these two subsets in the absence of IL-4, with a decrease of IL-4AC^+^ Th2 cells and a corresponding increase in IL-4AC^+^ Tfh cells (Figure 3B). Analysis of overall numbers shows that while the numbers of IL-4AC^+^ Tfh cells are not compromised by the lack of IL-4, the development of IL-4AC^+^ Th2 cells was inhibited by 50% in IL-4-deficient mice, illustrating partial dependence of this subset on IL-4 (Figure 3C). The MFI of IL-4AC expression was reduced in both IL-4AC^+^ Tfh and Th2 subsets from the IL-4^−/−^ mice (Figure 3D), suggesting that IL-4 positively regulates *Il4* expression in CD4^+^ T cells. By contrast, the IL-4AC^−^ Tfh and IL-4AC^−^ non-Tfh populations survived better in IL-4-deficient conditions (Figures S5A,B in Supplementary Material).

Thus, it appears that the number of IL-4AC^+^ Tfh cells in the lymph node is independent of IL-4, while Th2 CD4 cells in the ear lymph node are partially dependent on IL-4 for their development.

### IL-4 Is Required for the Development of IL-4AC- and IL-4AC/IL-13DR-Expressing Th2 Subsets, but Not IL-13DR-Expressing CD4^+^ T Cell Subset, in Ear Tissue

**Figure 4:**
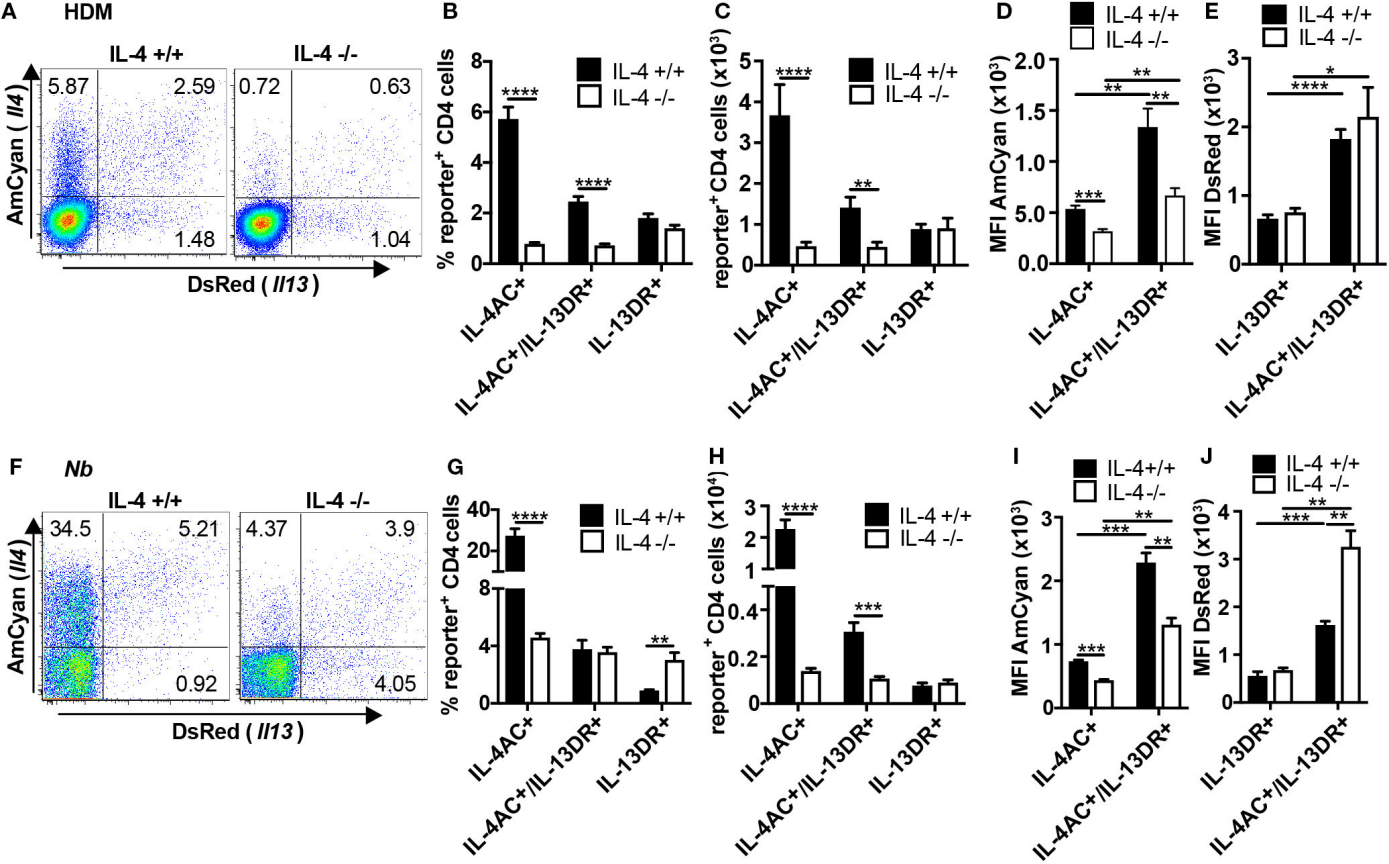
IL-4 is required for the development of IL-4AC- and IL-4AC/IL-13DR-expressing Th2 subsets but not the IL-13DR-expressing CD4^+^ T cell subset in ear tissue. 4C13R-IL-4^+/+^ and 4C13R-IL-4^−/−^ mice were treated with either **(A–E)** 200 μg house dust mite (HDM) or **(F–J)** 600 dead L3 *Nippostrongylus braziliensis* (*Nb*) i.d. in the ear pinnae. Ear tissue was harvested 7 days posttreatment. Tissues were analyzed to determine **(A,B,F,G)** proportion of IL-4AC^+^ and IL-13DR^+^ CD4 T cells, **(C,H)** number of IL-4AC^+^ and IL-13DR^+^ CD4 T cells, and **(D,E,I,J)** median fluorescent intensity (MFI) of IL-4AC and IL-13DR for each of the Th2 cell subsets. Data representative of seven experiments (*n* = 35–37) for HDM and two experiments (*n* = 6) for dead *Nb*. **(A)** FACS plots concatenated from a single representative experiment (*n* = 6). **(B,C)** Data pooled from seven experiments (*n* = 37). **(D,E)** Data from a single representative experiment (*n* = 6). **(F)** FACS plots concatenated from a single representative experiment (*n* = 3). **(G,H)** Data pooled from two experiments (*n* = 6). **(I,J)** Data from a single representative experiment (*n* = 3). Data show mean + SEM (*p ≤ 0.05, **p ≤ 0.01, ***p ≤ 0.001, and ****p ≤ 0.0001 two-tailed *t*-test).

We next studied the role of IL-4 in the development of Th2 subsets in the ear tissue following priming with either HDM or *Nb* allergens. As observed in Figure 1, three subsets of IL-4AC- or IL-13DR-expressing Th2 cells were identified in the ear after priming with HDM (Figures 4A,B) or dead *Nb* (Figures 4F,G). In both primary immunization models, the number of IL-13DR single-positive Th2 cells was not affected by the absence of IL-4 (Figures 4C,H), nor was there a reduction in the amount of IL-13DR they expressed as determined by MFI (Figures 4E,J). Although the proportion of IL-13DR Th2 cells was higher in IL-4^−/−^ mice primed with non-viable Nb compared with IL-4-sufficient controls (Figure 4G), analysis of the total cell numbers revealed no difference (Figure 4H), due to the reduced overall numbers of CD4^+^ T cells present in the IL-4^−/−^ mouse (data not shown), suggesting that the higher proportion was simply due to the lack of other cell subsets in the IL-4^−/−^ ear tissue. By contrast, the number of IL-4AC single-positive CD4 cells that appeared in the allergen primed ear tissue was reduced 8-fold in HDM immunized and an even greater 16-fold in *Nb* immunized 4C13R-IL-4^−/−^ mice (Figures 4C,H). The IL-4AC/IL-13DR double reporter-expressing Th2 subset was also dependent on IL-4, with a threefold reduction in their levels observed in 4C13R-IL-4^−/−^ mice in both models. Even though similar proportions of these cells were found in dead *Nb* immunized IL-4^+/+^ and IL-4^−/−^ mice (Figure 4G), their numbers were reduced in the IL-4-deficient mice due to the reduced level of CD4 T cells in these mice as mentioned earlier. Furthermore, the MFI of IL-4AC in the IL-4AC^+^ and IL-4AC^+^/IL-13DR^+^ Th2 cells was reduced by twofold in IL-4-deficient reporter mice (Figures 4D,I). Thus, as well as a significant reduction in the numbers of IL-4AC-expressing Th2 cells being detected in IL-4-deficient 4C13R-IL-4^−/−^ mice, the gene expression from the *Il4* locus, as determined by examining reporter expression, was also reduced. As previously noted with IL-4-sufficient mice (Figure 1G), dual reporter IL-4AC^+^/IL-13DR^+^ Th2 cells from 4C13R-IL-4^−/−^ mice also expressed greater levels of IL-4AC and IL-13DR reporters than the single reporter positive cells (Figures 4D,E,I,J).

To determine whether the distinct IL-4AC- and IL-13DR-expressing Th2 subsets could also develop at other tissue sites following antigen priming, we examined the Th2 subsets generated in the lung and BAL of mice responding to a live primary *Nb* infection, in which larvae infect the lung before migrating to the gut. We observed in both lung tissue and airways (BAL) the same three CD4^+^ IL-4AC-, IL-4AC/IL-13DR-, and IL-13DR-expressing Th2 subsets (Figures S6A,C in Supplementary Material). The appearance of the IL-4AC CD4^+^ Th2 subset was found to be significantly reduced in IL-4-deficient mice compared with IL-4-sufficient controls, the IL-4AC/IL-13DR and the IL-13DR CD4^+^ Th2 subset were not significantly affected by IL-4 deficiency (Figures S6B,D in Supplementary Material).

In summary, The IL-4AC- and IL-13DR-expressing Th2 subsets that appear in ear tissues following antigen priming show differential requirements for IL-4, with IL-4AC^+^ and IL-4AC^+^/IL-13DR^+^ Th2 cells being IL-4 dependent while the IL-13DR-expressing CD4 Th2 subset was not affected by the absence of IL-4.

### Reduction in IL-4 Availability Through *Il4* Gene Hemizygosity Has a Partial Effect on Development of IL-4AC- and IL-4AC/IL-13DR-Producing T Cell Subsets

**Figure 5:**
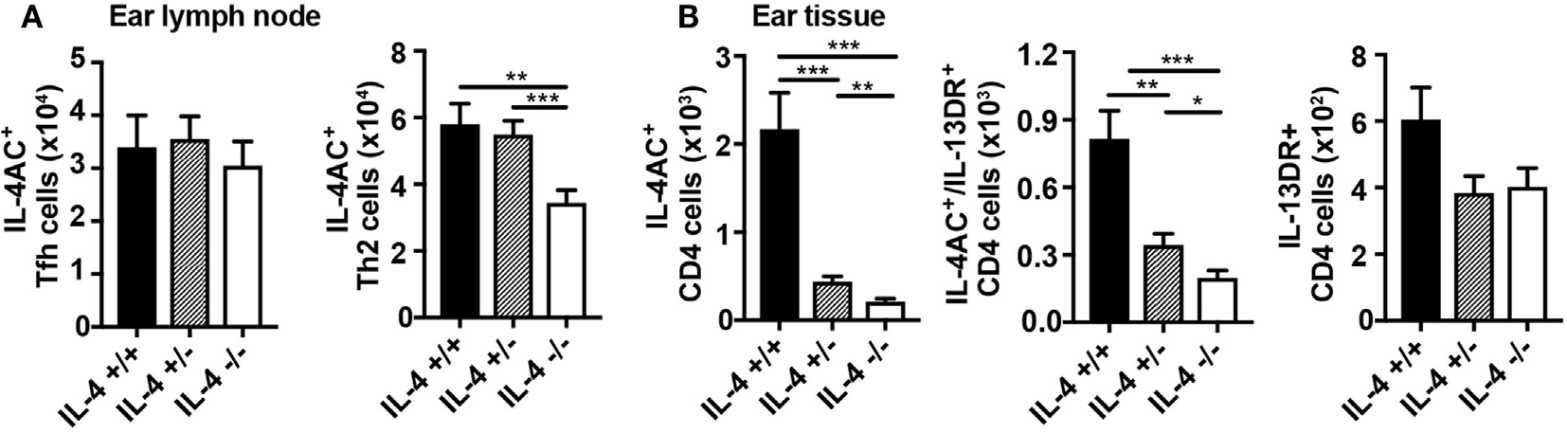
Reduction in IL-4 availability by *Il4* gene hemizygosity has a partial effect on the development of IL-4AC- and IL-4AC/IL-13DR-producing T cell subsets. 4C13R-IL-4^+/+^, 4C13R-IL-4^+/−^, and 4C13R-IL-4^−/−^ mice were treated with 200 μg house dust mite i.d. in the ear pinnae. Ear draining lymph nodes and ear tissue were harvested from the mice 7 days posttreatment. **(A)** Lymph nodes were analyzed to determine the number of IL-4AC^+^ T follicular helper (Tfh) and IL-4AC^+^ Th2 CD4 T cells. **(B)** Ear tissue was analyzed to determine the number of reporter^+^ Th2 CD4^+^ T cells. **(A)** Data pooled from three experiments (*n* = 18) for ear lymph node and **(B)** three experiments (*n* = 15) for ear tissue. Data show mean + SEM (*p ≤ 0.05, **p ≤ 0.01, and ***p ≤ 0.001 two-tailed *t*-test).

The profound effect of complete IL-4 deficiency on the development of IL-4AC- and IL-4AC/IL-13DR-expressing Th2 subsets in the skin tissue and lung, led us to wonder what would be the effect of a partial *Il4* gene deletion such as that seen in IL-4 hemizygous mice. IL-4-sufficient (4C13R-IL-4^+/+^), IL-4 hemizygous (4C13R-IL-4^+/−^), and IL-4-deficient (4C13R-IL-4^−/−^) mice were primed with HDM and their ear draining lymph nodes and ear tissue examined after 7 days. As reported in Figure 3, IL-4AC^+^ Tfh cells in the lymph node were not dependent on IL-4, whereas levels of IL-4AC^+^ Th2 cells were halved when IL-4 was not available (Figure 5A). Contrary to the IL-4-deficient system, the number of IL-4AC^+^ Th2 cells in the hemizygous lymph nodes were the same as those in IL-4 wild-type mice indicating that even with reduced IL-4 availability the IL-4AC^+^ Th2 cell response to HDM in the draining lymph node is capable of full development. In the tissue, however, the hemizygous *Il4* condition had a far more striking effect on the development of the IL-4AC^+^ Th2 subset, with a fivefold reduction in cell numbers to levels only twofold greater than in the absence of IL-4 (Figure 5B). The reduction of the IL-4AC^+^/IL-13DR^+^ Th2 subset in the ear was less pronounced and, as was seen in Figure 4, the IL-13DR^+^ Th2 cells were independent of IL-4 for their appearance in ear tissue. The disproportionate effect that the loss of half the wild-type IL-4 has on the IL-4AC^+^ and the IL-4AC^+^/IL-13DR^+^ Th2 subsets in the ear reinforces the importance of IL-4 in the development of this distinct CD4 Th2 effector subset in the tissue microenvironment of the skin.

## Discussion

We sought to clarify the role of IL-4 in the differentiation, migration, and accumulation of CD4^+^ T cells following a primary response to allergen. We followed the number of IL-4- and IL-13-expressing CD4 T cells that appear in the draining lymph node and subsequent type 2 inflammatory response in the skin or lung following intradermal priming with allergen or parasite infection, respectively. We confirm that during the 7-day priming period the appearance of IL-4-expressing Tfh cells in the lymph node is not dependent on IL-4, while the appearance of IL-4-expressing Th2 cells in the lymph node is partially affected by the absence of IL-4. We describe for the first time the profound requirement for IL-4 in regulating the appearance of IL-4- and IL-4/IL-13-expressing Th2 subsets in the skin tissue 7 days following allergen priming. A similar IL-4 response profile was found for IL-4-producing Th2 cells in the lungs of *Nb* challenged mice. Surprisingly, we identified a unique CD4 T cell subset in the skin that was induced by both allergen priming in the skin and parasite infection in the lung that was committed solely to IL-13 expression and was completely independent of IL-4. Although normally IL-13 expression in CD4 T cells is linked to IL-4 expression and it is viewed as one of the canonical Th2 cytokines which is regulated by IL-4, a previous study using IL-4/IL-13 reporter mice identified that IL-13 expression occurs in lung tissue but not lymph nodes and that IL-4 and IL-13 expression is not always linked (23). Here, using our HDM skin priming model, we complement this data with confirmation in the ear skin tissue and its draining lymph node of the confinement of IL-13-expressing Th2 cells to the tissue compartment, while IL-4-expressing Th2 subsets reside in both lymphoid and non-lymphoid tissues. Furthermore, our finding that *in vitro* restimulation did not elicit IL-13 expression in CD4 T cells derived from the draining lymph node of HDM ear immunized mice confirms that the failure to detect IL-13 is not a sensitivity issue of the reporter system but rather whether the CD4 T cells have received the appropriate signals for activation of the *Il13* gene. The identification in the tissues of primed mice of a unique subset of CD4 T cells that only expressed IL-13 and whose appearance was independent of IL-4 was surprising. When viewed in the context of recent work by others (8, 24) and our recent data showing that TSLP can regulate the development of an IL-13-producing CD4 Th2 subset (25), it becomes clear that other factors such as tissue alarmins are likely able to regulate or act as a tissue checkpoint for a IL-13-producing Th2 subset in the tissues.

T follicular helper cells have been shown to be a significant source of IL-4 in the lymph nodes during the Th2 response (21). In our studies following HDM priming of the ear skin, we find that both Tfh and Th2 cells contribute to IL-4 expression in the ear lymph node, although the Tfh cells had a greater degree of activation and made a greater amount of IL-4 than their Th2 counterparts, perhaps to aid their role helping B cells to class switch to IgE and IgG1 production. Tfh cells are traditionally thought to reside in the B cell follicle of the lymph nodes, but recent research has suggested that additionally Tfh cells may be precursors of Th2 cells subsequently found in the tissues (26, 27). Using a multiple prime and challenge lung model, Ballesteros et al. showed that HDM sensitization induced IL-4-committed Tfh cells in the lymph node which developed into IL-4- and IL-13-producing effector Th2 cells in the lung upon HDM challenge (26). Other research has identified a subset of Th2 promoting IL-21-expressing Tfh cells, distinct from Th2 cells, found in both the lymph node and lung tissue (27). Whether or not the Tfh cells seen in the ear lymph node in our ear HDM priming model are distinct from the ear tissue Th2 subsets remains to be determined. In addition, the link between the IL-4^+^ Th2 subset seen in the lymph node and those Th2 subsets in the ear is unclear and subject to further research. It is probable that IL-4-expressing Th2 cells migrate from the lymph node to the ear tissue where they produce additional Th2 cytokines such as IL-5 and IL-13 for Th2 effector functions, perhaps under response to local damage elicited tissue signals (8). Of the IL-4-producing CD4^+^ subsets in the lymph node, the Th2 subset is more likely than the Tfh cells to be the precursors of the tissue Th2 subsets, as both the Th2 subsets, but not Tfh cells demonstrate a dependence on IL-4 for their development. It should be noted though that the MFIs of the IL-4AC reporter were significantly reduced in both the Tfh and Th2 lymph node CD4 T cells subsets indicating that the levels of IL-4 may be regulated through indirect means such as signaling by B cells or dendritic cells.

That the development of IL-4-expressing Tfh cells is independent of IL-4 adds to current recognition of Tfh cells being a unique subset distinct from traditional Th2 cells. Development of Tfh cells instead depends on IL-6, IL-21, STAT3, and BCL6 and not on other cytokine or transcription factors necessary for formation of other T helper subsets (28–30), and it has been reported that the Tfh cell transcription factor BCL6 can limit the activities of Th2 cells (31). However, it should be noted that studies by others show that the IL-4-producing Th2 subset in the lymph node is not affected by BCL6 deletion indicating that the Tfh subset may not be a precursor for lymph node Th2 (32). Our studies identify a second IL-4-expressing CD4 T cell subset in the lymph node that does not express Tfh markers but whose expression of IL-4 appears to be partially IL-4 dependent. In support of our finding, recent studies have shown that IL-4Rα knockout mice exhibited a significantly compromised IL-4-expressing Th2 response in the gut draining mesenteric lymph node of *H. polygyrus*-infected mice (33) As IL-4Rα is a component of the receptors *via* which both IL-4 and IL-13 act, this could be attributed to the inability of IL-4 (or IL-13) to act *via* its receptor. In our studies, generation of IL-4AC-expressing lymph node Th2 cells was not effected in mice lacking one IL-4 allele and was only reduced by half in the complete absence of IL-4, thus showing only a partial requirement for IL-4. This suggests that although IL-4 contributes to Th2 differentiation in the lymph node other factors are likely involved, such as the quality of the TCR signal and co-stimulation, Th2 promoting miRNAs, signaling pathways that promote GATA3 expression, and other Th2 promoting cytokines like TSLP, IL-33, and IL-25 (34–39). In contrast to our present findings, previous research using G4 reporter mice has indicated that IL-4 was not required to generate IL-4-expressing Th2 cells in the lymph nodes of *Nb*-infected mice (6). However, in these studies only G4/IL4 heterozygous mice in which IL-4 was produced from only one allele could be compared with IL-4-deficient G4/G4 mice in the various allergen and parasitic models and no difference in numbers of GFP-expressing Th2 cells was seen. The use of our dual IL-4, IL-13 4C13R reporter mice in this study, where the reporters are inserted into a bacterial artificial chromosome leaving the endogenous cytokines intact, has enabled a fuller comparison of the effect of the IL-4-sufficient, IL-4 heterozygous, and the IL-4-deficient states on IL-4-expressing CD4 T cell development. In agreement with the previous work, no significant difference was seen between numbers of Th2-differentiated cells in IL-4^+/−^ and IL-4^−/−^ mice in the lymph node, but the additional comparison with the IL-4^+/+^ mice enabled the conclusion that differentiation of Th2 cells is in fact partially dependent on IL-4. Other research comparing NP-OVA/Alum ear immunized IL4^+/+^, IL4^+/−^, and IL-4^−/−^ 4C13R mice also showed IL-4-expressing Th2 cells in the lymph node to not be dependent on IL-4 (20). In another study investigating IL-4- and IL-13-expressing cells arising in response to a model of OVA-induced lung allergy (40), dual reporter *Il4*^+/eGFP^*Il13*^+/Tom^ mice were used, which due to the insertion of the reporter constructs, are hemizygous for the IL-4 and IL-13 cytokines. They found that IL-4eGFP-expressing CD4^+^ T cells were largely absent from the lung while in the mediastinal lymph node they were present and major contributors to IL-4 production. This replicates what we have seen in IL-4 hemizygous mice in our current study (Figure 5), where IL-4AC^+^ Th2 cells are not sensitive to the partial loss of IL-4 in the lymph node but in the ear tissue are sensitive to even the hemizygous IL-4 state, with severely decreased numbers. The reliance on IL-4-knockin reporter mice fails to achieve an accurate representation of cytokine expression in a fully IL-4-sufficient environment, while the 4C13R reporter mice overcome this limitation. However, it should be noted that studies using gene expression reporter constructs do not necessarily give any indication of the protein levels that are produced by these Th2 cells and further studies would be required to determine whether the gene expression findings reported here translate into protein expression.

**Figure 6:**
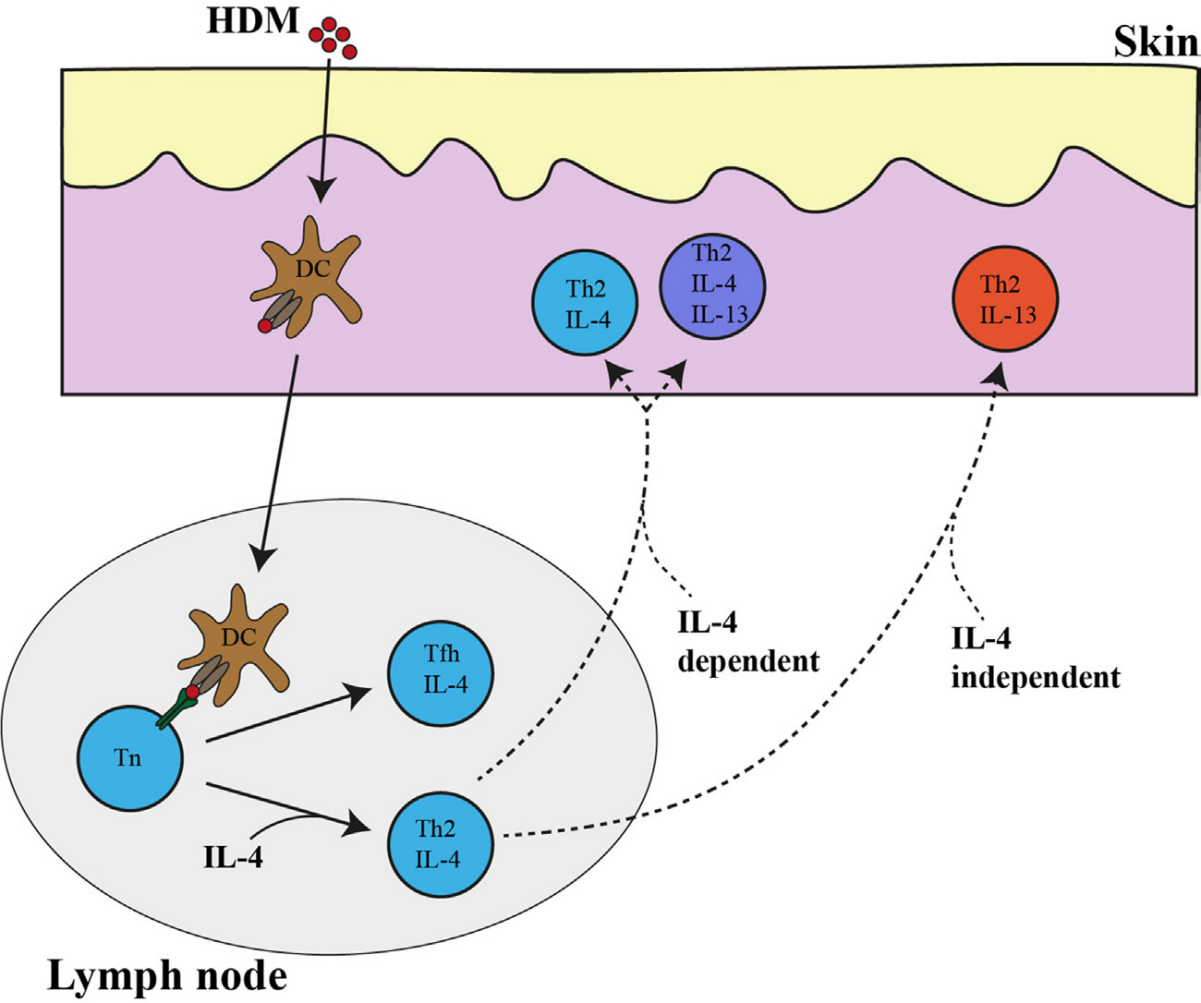
IL-4- and IL-13-expressing Th2 subsets in the lymph node and ear tissue after allergen priming. Following house dust mite (HDM) priming in the ear skin, dendritic cells take up allergen and transport it to the ear draining lymph node where they present it to naïve CD4 T cells. Naïve CD4 T cells differentiate into either (i) IL-4-expressing Th2 cells in a process that is partially dependent on IL-4 or (ii) IL-4 independent IL-4-expressing T follicular helper (Tfh) cells. IL-4 single-positive CD4 T cell and IL-4/IL-13 double-positive-expressing CD4 T cell subsets in the skin tissue are very dependent on IL-4 while IL-13 single-positive-expressing Th2 cells are independent of IL-4. The broken line represent the as of yet unproven but potential link between the lymph node Th2 cells and those in the tissue.

In addition to the IL-4-expressing Tfh subset, we have identified using IL-4AC and IL-13DR expression three subsets of CD4^+^ T cells that would fall under the definition of a Th2 effector cell that is primed by allergen (or parasite antigens) in the draining lymph node and then migrate and expresses functional cytokines at the original antigen challenged tissue site. The IL-4- and IL-4/IL-13-expressing Th2 subsets are very dependent on IL-4 for their appearance in the tissues. Although outside the scope of this study, the requirement for IL-4 may be either to (i) allow differentiation/survival of differentiating Th2 subsets to *Il4* and *Il13* gene expression, (ii) regulate migration and extravasation of CD4 Th2 subsets to antigen challenged tissue sites or (iii) its continued presence is required when reactivated with antigen at the tissue site for full differentiation to a IL-4- or IL-4/IL-13-expressing Th2 effector to occur. Also, our observation that a distinct IL-13-expressing CD4 T cell subset appears in tissues following antigen challenge that is not regulated by IL-4 may reflect the ability of specific tissue-derived alarmins to program specific cytokine expression patterns. Taken together, a picture emerges whereby the differentiating Th2 cell appears to be regulated by multiple checkpoints at distinct tissue sites (Figure 6). The lymph node appears to be a place for antigen priming and activation of naïve T cells to Th2 precursors, some of which sequester to the B cell areas of the lymph node to drive B cell differentiation and the others to quickly circulate throughout the body and home to the tissue sites challenged with priming antigens. IL-4 and specific tissue-derived alarmins and factors appear able at this point to direct the further differentiation of the Th2 precursors to distinct subsets of IL-4-, IL-4/IL-13-, and IL-13-expressing subsets. The value or relevance of each Th2 effector subset to the process of antigen/pathogen neutralization, clearance, and repair needs to be elucidated by further studies.

The development of reagents which can selectively interfere with the actions of Th2 cytokines is a potential therapeutic approach in the treatment of allergic disorders, and antibodies targeting the individual cytokines and their receptors have met with variable results [reviewed in Ref (41)]. Dupilumab that blocks IL4Rα, a subunit of both the IL-4 and IL-13 receptors, interferes with the action of both these cytokines and to date has had success in treating asthma and atopic dermatitis (42, 43). The ability of this drug to simultaneously target and block the functions of the lymph node and tissue Th2 cells, thus inhibiting humoral and cellular aspects of type 2-driven pathology is likely key to its success. Further insights into the nature of IL-4- and IL-13-producing Th2 subsets generated in response to allergic stimuli will beneficially further the understanding of the anti-allergic effects of these agents and contribute to their ongoing development.

## Ethics Statement

All animal procedures were approved by the Victoria University of Wellington Animal Ethics committee and performed in accordance with institutional guidelines.

## Author Contributions

MP carried out the experiments, analyzed the data, and wrote the manuscript. GG, FR, and RK provided conceptual insights and editing of the manuscript and GLG supervised the project. All authors provided feedback on the manuscript.

## Conflict of Interest Statement

The authors declare that the research was conducted in the absence of any commercial or financial relationships that could be construed as a potential conflict of interest.
