# Supplementary material for: IL-4 Is a Key Requirement for IL-4- and IL-4/IL-13-Expressing CD4 Th2 Subsets in Lung and Skin
Source: Front Immunol. 2018 Jun 1;9:1211. doi: 10.3389/fimmu.2018.01211 (PMC5992292; doi:10.3389/fimmu.2018.01211)
Supplement: Supplementary file 1 [file data_sheet_1.DOCX]

Supplementary Material

**IL-4 is a key requirement for IL-4 and IL-4/IL-13 expressing CD4 Th2 subsets in non-lymphoid tissues.**

**Melanie Prout, Ryan L. Kyle, Franca Ronchese, Graham Le Gros^*^**

*** Correspondence:** Corresponding Author: [glegros@malaghan.org.nz](mailto:glegros@malaghan.org.nz)

**Supplementary Figure 1. Kinetics of induction of IL-4AC^+^ CD4 T cells in the ear lymph node after i.d. ear priming with HDM.**

4C13R and C57BL/6 mice were primed with 200ug HDM i.d. in the ear and the ear draining lymph nodes examined days 0, 2, 3, 5, 7, 9, 11, 13 and 17 post treatment by flow cytometry to determine **(A)** the proportion of IL-4AC expressing CD4 T cells and **(B)** the number of IL-4AC expressing CD4 T cells. Data from a single experiment (n=4).

**Supplementary Figure 2. Gating strategy for CD4^+^ T cells in the ear tissue and ear lymph node.**

Strategy used for gating on **(A)** ear tissue and **(B)** ear lymph node CD45^+^ cells in experiments involving HDM or dead *Nb* treatment of 4C13R mice.

**Supplementary Figure 3. Priming with *Nb* allergen induces the development of Th2 subsets expressing only IL-4AC in the lymph node and distinct IL-4AC^+^, IL-4AC^+^/IL-13DR^+^ or IL-13DR^+^ expression profiles in the ear tissue.**

4C13R transgenic mice were primed with 600 dead L3 *Nb* i.d. in the ear. Ear draining lymph nodes and ear tissue were harvested 7 days later and the presence of IL-4AC and IL-13DR expressing CD4 Th2 cells examined by flow cytometry.  **(A, C)** Number of CD4 T cells in ear lymph node and ear tissue. **(B, D)** Concatenated FACs plots of CD4 T cells from naïve and *Nb* primed 4C13R transgenic mice showing IL-4AC ^+^ and IL-13DR ^+^ cells. **(E)** Proportions and **(F)** numbers of IL-4AC ^+^, IL-4AC ^+^/IL-13DR^+^ and IL-13DR^+^ subsets in the ear lymph node and ear tissue. **(G)** Median fluorescent intensity (MFI) of IL-4AC and IL-13DR reporters expressed in single reporter^+^ vs double reporter^+^ CD4 Th2 cells in the ear tissue (relative to MFI of single positive cells). **(A, C, E, F, G)** Data pooled from 3 experiments (n=26) for ear lymph node and 2 experiments (n=6) for ear tissue **(B)** Facs plots concatenated from a single representative experiment (n=11). **(D)** Facs plots concatenated from a single representative experiment (n=3). Data shows mean + SEM. **p≤0.01, ***p≤0.001 ****p≤0.0001 two-tailed *t*-test.

**Supplementary Figure 4. IL-4AC^+^ Th2 cells in the lymph node do not express IL-13DR upon *in vitro* stimulation.**

Ear draining lymph node cells were isolated from mice 7 days after HDM immunisation and whole lymph node cells re-stimulated for 19 hours with plate bound anti-CD3 + anti-CD28 + IL-2. Proportion of CD4 T cells expressing **(A)** IL-4AC, **(B)** IL-13DR or **(C)** IL-4AC/IL-13DR. Results from a single *in vitro* experiment (n=11). Data shows mean + SEM.

**Supplementary Figure 5 IL-4AC^-^ Tfh and IL-4AC^-^ non-Tfh cells in the lymph node exhibit enhanced survival in the absence of IL-4.**

4C13R-IL-4^+/+^ and 4C13R-IL-4^-/-^ mice were treated with 200ug HDM i.d. in the ear. The ear draining lymph nodes were harvested from mice 7 days post treatment and lymph nodes analysed by flow cytometry. **(A)** FACs plots showing the gating of IL-4AC^-^ CD4 T cells and then the proportion of CXCR5^+^ PD1^+^ Tfh and CXCR5^lo^ PD-1^lo^, CXCR5^+^ PD-1^lo^ and CXCR5^lo^ PD-1^+^ CD4^+^ non-Tfh cells within this population. **(B)** Numbers of IL-4AC^-^ CD4 T cells that are Tfh or non-Tfh cells. **(A)** FACS plots from a representative experiment (n=6). **(B)** Data pooled from 3 experiments (n=18). Data shows mean + SEM. *p≤0.05 **p≤0.01, ***p≤0.001 two-tailed *t*-test.

**Supplementary Figure 6. IL-4 is required for the development of IL-4AC and IL-4AC/IL-13DR expressing Th2 subsets but not IL-13DR only subsets in the BAL and Lung tissue.**

4C13R-IL-4^+/+^ and 4C13R-IL-4^-/-^ mice were infected with 550 L3 *Nb* s.c. **(A, B)** Lung and **(C, D)** BAL were harvested 7 days post infection. Tissues were analysed to determine **(A, C)** proportion of reporter^+^ CD4 T cells and **(B, D)** number of reporter^+^ CD4 T cells. Data from a single experiment (Lung n=10 and BAL n=3). Facs plots concatenated from this experiment. Data shows mean + SEM. *p≤0.05 two-tailed *t*-test.
